# HIV-associated Burkitt lymphoma with bone marrow and cerebral invasion in a patient with history of *Plasmodium falciparum* infection

**DOI:** 10.1186/1471-2334-14-S4-P17

**Published:** 2014-05-29

**Authors:** Irina Lăpădat, Cristina Popescu, Raluca Dulamă, Alina Lobodan, Anca Ruxandra Negru, Mihaela Rădulescu, Violeta Molagic, Ruxandra Moroti, Cătălin Tilişcan, Victoria Aramă

**Affiliations:** 1 National Institute for Infectious Diseases “Prof. Dr. Matei Balş”, Bucharest, Romania; 2 Carol Davila University of Medicine and Pharmacy, Bucharest, Romania

## 

HIV infected patients are more likely to develop non-Hodgkin lymphoma (NHL). Burkitt lymphoma (BL) is a highly aggressive NHL, associated with immunosuppression, especially with HIV. According to WHO classification there are three clinical types of BL: endemic, sporadic and immunosuppression-associated. Sporadic lymphoma was described especially in children (40% of child lymphomas in USA and EU). Two cofactors seem to be associated with BL: Ebstein Barr virus (EBV) and *Plasmodium falciparum* (PF) infection. PF and EBV are well-known co-factors in the pathogenesis of BL, but the mechanisms of interaction remain unclear.

We present a 51 year-old male, who developed lymphadenopathy, prolonged fever, weight loss, splenomegaly and seizure. The patient was admitted to a Hematology University Hospital. After lymph node biopsy he was diagnosed with BL. A specimen of bone marrow from the right iliac crest showed gross invasion by Burkitt tumor cells.

The patient tested positive for HIV and he was referred to the National Institute of Infectious Diseases “Prof. Dr. Matei Balş”, Bucharest. According to CDC Classification System for HIV Infection the patient had AIDS (C3 stage with a CD4 count of 39/cmm) and a high HIV viral load (500,262 copies/mL). The patient’s medical history revealed *Plasmodium falciparum* malaria 4 years ago, while he was living in South America. Epidemiological data revealed more than 200 sexual partners in the last two years. At admission to our hospital he had pancytopenia: white blood cells 1400/cmm, with 600 neutrophils and 600 lymphocytes, hemoglobin 9.6 g/dL and platelet count 25,000/cmm. The patient was tested for EBV infection and high titer of anti-VCA antibodies was found. ART was initiated with TDF-FTC-lopinavir/r with good virological outcome (after 6 weeks of therapy the viral load was 383 copies/mL). Cerebral MRI showed diffuse lymphomatous invasion. After 3 weeks of ART the patient was referred to Hematology Hospital where chemotherapy was started. Post-chemotherapy the pancytopenia was more severe: white blood cells – 200/cmm with CD4 count – 10/cmm, platelets – 15,000/cmm and hemoglobin – 7.9 g/dL. The BL response after chemotherapy was poor and the patient died two months after the diagnosis, despite the good virological outcome.

We present a rare case of NHL in a HIV-infected patient, with multiple co-factors for BL: HIV infection, EBV infection and *Plasmodium falciparum* infection. The prognostic in AIDS depends on the comorbidities’ outcome.

